# Osteocalcin serum concentrations and markers of energetic metabolism in pediatric patients. Systematic review and metanalysis

**DOI:** 10.3389/fped.2022.1075738

**Published:** 2023-01-12

**Authors:** Silvia Rodríguez-Narciso, Raigam Jafet Martínez-Portilla, Iris Paola Guzmán-Guzmán, Gabriela Careaga-Cárdenas, Brenda Jazmin Rubio-Navarro, Luis Fernando Barba-Gallardo, Rodolfo Delgadillo-Castañeda, José Rafael Villafan-Bernal

**Affiliations:** ^1^ Basic Sciences Center, Autonomous University of Aguascalientes, Aguascalientes, Mexico; ^2^ Clinical Research Division, Evidence-Based Medicine Department, National Institute of Perinatology, Mexico City, Mexico; ^3^ Molecular and Maternal-Fetal Medicine, Iberoamerican Research Network in Translational, Mexico City, Mexico; ^4^ Faculty of Chemical-Biological Sciences, Universidad Autónoma de Guerrero, Chilpancingo, Mexico; ^5^ Center for Health Sciences, Autonomous University of Aguascalientes, Aguascalientes, Mexico; ^6^ Department of Pediatrics, Centenario Hospital Miguel Hidalgo, ISSEA Aguascalientes, Aguascalientes, Mexico; ^7^ Investigador por México, National Council of Science and Technology (CONACYT), Mexico City, Mexico; ^8^ Laboratory of Immunogenomics and Metabolic Diseases, Mexican National Institute of Genomic Medicine (INMEGEN), Mexico City, Mexico

**Keywords:** osteocalcin, energetic metabolism, glucose - insulin, pediatric patient, systematic review & meta-analysis

## Abstract

**Background:**

Osteocalcin plays a role in glucose metabolism in mice, but its relevance in human energetic metabolism is controversial. Its relationship with markers of energetic metabolism in the pediatric population has not been systematically addressed in infants and adolescents.

**Objective:**

This study aims to assess the mean differences between tOC, ucOC, and cOC among healthy children and children with type 1 or type 2 diabetes (T1D or T2D) and the correlation of these bone molecules with metabolic markers.

**Methods:**

A systematic review and metanalysis were performed following PRISMA criteria to identify relevant observational studies published in English and Spanish using PubMed, Scopus, EBSCO, and Web of Science databases. The risk of bias was assessed using New Castle–Ottawa scale. Effect size measures comprised standardized mean difference (SMD) and Pearson correlations. Heterogeneity and meta-regressions were performed.

**Results:**

The 20 studies included were of high quality and comprised 3,000 pediatric patients who underwent tOC, cOC, or ucOC measurements. Among healthy subjects, there was a positive correlation of ucOC with WC and weight, a positive correlation of tOC with FPG, HDL-c, WC, height, and weight, and a negative correlation between tOC and HbA1c. Among diabetic subjects, a negative correlation of ucOC with HbA1c and glycemia in both T1D and T2D was found and a negative correlation between tOC and HbA1c in T1D but not in T2D. The ucOC concentrations were lower in T2D, T1D, and patients with abnormal glucose status than among controls. The serum concentrations of tOC concentrations were lower among T1D than in controls. The patient's age, altitude, and HbA1c influenced the levels of serum tOC.

**Conclusion:**

Osteocalcin is involved in energy metabolism in pediatric subjects because it is consistently related to metabolic and anthropometric parameters.

**Systematic Review Registration:**

https://www.crd.york.ac.uk/prospero/, identifier: CRD42019138283.

## Introduction

After the discovery of the effects of undercarboxylated osteocalcin (ucOC) in regulating glucose metabolism, insulin release, and insulin sensibility in mice (1), several studies in human have confirmed the relevance of this bone-derived hormone in the regulation of glucose metabolism in human adults (2–6).

In adults, previous metanalysis demonstrated significant differences in total osteocalcin (tOC) serum concentrations between type 2 diabetics (T2D) and healthy subjects (HS) (7, 8). Women with gestational diabetes exhibit significant differences in tOC serum levels compared to normal pregnant (9). However, the role of this hormone in regulating glycemia and insulin sensibility in children and adolescents has been briefly studied (10, 11), and no metanalysis formerly analyzed the role of osteocalcin in energetic metabolism nor its relationship to other metabolic parameters in this population.

During infancy and adolescence, osteocalcin serum levels are higher because of the highest osteoblast activity necessary to support bone growth and the high rate of bone remodeling (12, 13). Consequently, the osteocalcin serum levels increase to parallel the growth velocity curve (14).

Although some original studies report altered serum levels of tOC in type 1 diabetes mellitus (T1D) (15, 16), few small studies have measured ucOC and cOC (two specific types of osteocalcin) in T1D (17). Furthermore, no clarity exists on the association of ucOC with energetic metabolism markers in healthy and T2D children. Since ucOC is claimed to be the osteocalcin type with a role in the modulation of energetic metabolism (18, 19), it is necessary to clarify the role of this hormone in metabolism in human children and adolescents.

In the present study, we analyze if there are significant differences in osteocalcin serum concentrations (cOC, ucOC, and total OC) between healthy and diabetic pediatric patients. In addition, we estimated the correlation of osteocalcin with markers of energetic metabolism in pediatric subjects with normal glycemic status and with T1D or T2D.

## Material and methods

### Protocol registration

This project was registered in the PROSPERO International prospective register of systematic reviews (registration number: CRD42019138283).

### Eligibility criteria, information sources, and search strategy

A systematic search was executed in PubMed, Scopus, Web of Science, The Cochrane Library, and PROSPERO databases to identify relevant studies published in English and Spanish restricted to human with no restriction in publication date. The query and keywords of the search are presented in Supplementary S1A. additional manual search was conducted to identify additional relevant publications. The first search was run on July 20, 2018. Afterward, the update was extended until March 20, 2022. This review was carried out adhering to the Meta-analysis of Observational Studies in Epidemiology (MOOSE) guidelines (20), and the Preferred Reporting Items for Systematic Reviews and Meta-Analyses (PRISMA) guidelines for systematic reviews and meta-analysis (21, 22). Two independent evaluators evaluated the abstracts (GCC & BJRN), blinded to authorship, authors' institutional affiliation, and study results. If the abstract fulfilled the inclusion criteria, full-text articles were then reviewed. The third and fourth investigators (JRVB & RJMP) independently resolved any disagreement between evaluators. Corresponding authors were reached by e-mail to request such data in the case of relevant studies with missing information.

### Study selection

We include in this systematic review and meta-analysis only observational cross-sectional studies that included pediatric patients (less than 18 years old) with T1D, T2D, or nondiabetic controls reporting mean serum levels of osteocalcin (cOC, ucOC or tOC) and/or coefficient correlations between any type of osteocalcin (cOC, ucOC or tOC) and fasting glucose, fasting insulin, HOMA-IR, HabA1c, HDL-c, LDL-c, body mass index (BMI), waist circumference (WC), height, weight, and age. We excluded studies with no information on mean levels of osteocalcin or the previously mentioned correlations. Nondiabetic controls were those groups of individuals reported as healthy by the authors of the primary studies, namely subjects without type 1, type 2 diabetes, or carbohydrate intolerance.

### Data extraction

The following information was extracted using a datasheet based on Cochrane Consumers and Communication Review Group's data extraction template (23): author, year of publication, the country where the study was conducted, type of study, original inclusion and exclusion criteria, diabetes type, type of osteocalcin, the method for measurement of osteocalcin, intra and inter-assay coefficient of variation, the total number of patients included in the study, the total number of participants, mean BMI and age, gender, method of quantification and brand of the kit used for the measurement. Additionally, information about the mean UV index and altitude (as surrogates of sun exposure) of the city where the study was conducted was obtained from Weather Government Agencies of each Country.

### Assessment of risk of bias

Two reviewers (FEPM & RDC) independently assessed the quality of the selected studies. In case of disagreement in the evaluation, a third researcher resolved it (JRVB or RJMP). Quality assessment of observational studies was carried out using the Newcastle–Ottawa Scale for cross-sectional studies. Each study was judged on three dimensions: the study groups' selection, the groups' comparability, and the ascertainment of exposure. One star was given for each signaling question among each dimension. The total number of possible stars were nine, and studies with six or more stars were considered high-quality, while studies with less than six were considered low-quality (24).

### Data analysis

Before analysis, all osteocalcin values were converted to the same unit of measurement. Then extracted quantitative data were pooled in the metanalysis. For data analysis, the recommendations of Cochrane Handbook were followed as described: ucOC and tOC were compared between T1D or T2D and healthy controls. The effect size was expressed as standardized mean difference (SMD) by random-effects model (REM) weighted by the inverse of the variance since all studies used randomly sampled (25). Results are presented using forest plots of SMDs and Pearson correlations for the main groups (T1D or T2D and controls). Inter-study variability was assessed using the *τ*^2^, Cochran's *Q*, and *I*^2^ statistics (26). The contribution of individual study heterogeneity was visually assessed by Baujat plots (25).

Additionally, the effect size was determined for correlations (Pearson or Spearman) of tOC, ucOC, or cOC with fasting glucose, fasting insulin, HOMA-IR, HabA1c, HDL-c, LDL-c, body mass index (BMI), waist circumference (WC), height, weight, and age.

Since enough information was available only for tOC, univariate and multiple meta-regressions were performed to add another approach for unexplained heterogeneity and to determine which variables influenced tOC serum levels. The following covariates individually or combined were used: patient age, altitude, UV index, HbA1c, and method for tOC quantification. *I*^2^ and *R*^2^ values were reported to present residual heterogeneity and the amount of heterogeneity explained by each variable or by the multiple meta-regression model. Residual analysis was performed to test the validity of the multiple meta-regression model.

Publication bias was visually assessed by contour-enhanced funnel plots and quantified by Egger method. Moreover, a cumulative analysis was performed and presented as a forest plot to assess an “small study effect” defined as the chance of finding a trend towards a larger effect due to the higher probability of a small study of being published when a more “significant” result is found (27, 28). Statistical analysis was conducted using R studio v1.1.463 (R Foundation for Statistical Computing) [package “meta v4.2”].

## Results

### Study selection and study characteristics

**Figure 1:**
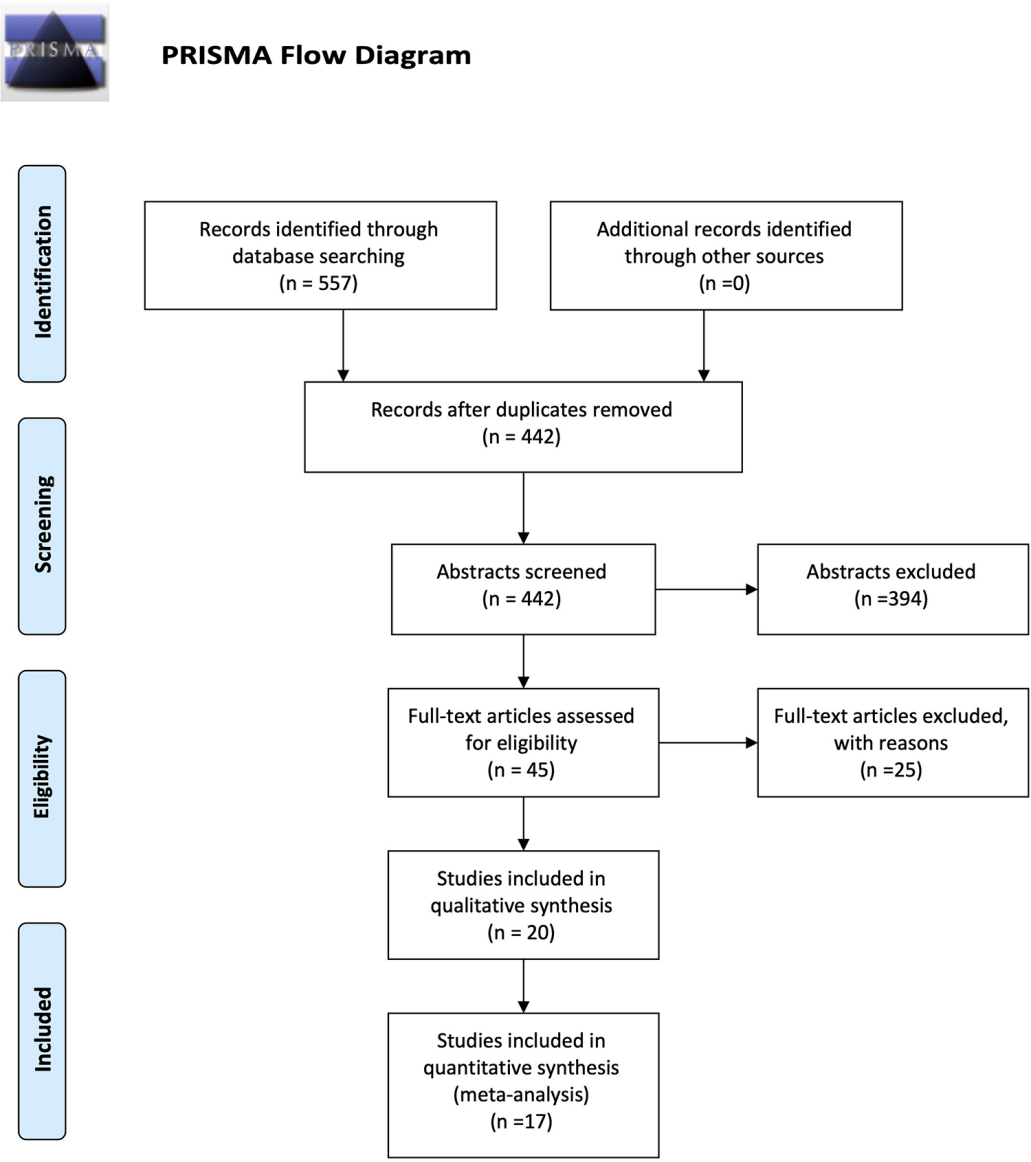
PRISMA flow diagram of the study.

A total of 557 studies were identified by database searching, and 45 were eligible for full-text review. After review, 20 studies were retained for the systematic review and 17 for meta-analysis. Specific reasons for excluding 25 full-text studies are presented in Supplementary S1B, including no existence of a control group, the inclusion of adults and children (mixed population) in the same analysis, or with diabetic ketoacidosis. Furthermore, studies with no observational design or failure to report osteocalcin serum levels were excluded (Figure 1). Some authors were reached by email to obtain missed data about their article, but no answer was achieved. The general characteristics of the included articles are described in Table 1 (10, 11, 15–17, 29–45).

**Table 1:** General characteristics of the included studies.

Author	Year	Country	Type of study	Inclusion criteria	Exclusion criteria	Diabetes type	Type of osteocalcin	Analysis method	Intra-interassay CV	*N* study	Age at inclusion	Gender %M/F
Faienza	2018	Italy	Cross-sectional	Age greater than 5 years, and disease duration of more than 2 years	Use of vitamin or mineral supplements or dietary restrictions, chronic diseases with a possible impact *n* bone metabolism and the use of medications affecting bone metabolism	T1D	Total OC	EIA	NR	131	11	47/53%
Giudici	2017	Brazil	Cross-sectional	Adolescents between 14–18 years living in Sao Paolo	Chronic disease besides obesity	Healthy	Total and ucOC	ELISA	NR	198	16.3	51/49%
Huneif	2017	Saudi Arabia	Cross-sectional	Type 1 DM by WHO criteria	Diabetic children treated with warfarin, heparin, drugs for osteoporosis, vit D, glucocorticoids, anticonvulsant drugs, other types of DM, renal, liver and thyroid disease	T1D	Total OC	ELISA	NR	204	4-14	57/43%
Redondo	2017	EUA	Prospective	Children with new-onset diabetes between 2010–2012 with data cOC, uOC	NR	T1D, T2D	ucOC and cOC	EIA	NR	70	11.8	49/51%
Takaya	2017	Japan	Cross-sectional	Diabetic and obese subjects without macrovascular disease or microalbuminuria	NR	T2D	ucOC, OC	ELISA	2.6%	39	13.5	66/34%
Faienza	2017	Italy	Cross-sectional	Age greater than 5 years, and disease duration of more than 2 years	Use of vitamin or mineral supplements or dietary restrictions, chronic diseases with a possible impact *n* bone metabolism and the use of medications affecting bone metabolism	T1D	Total OC	EIA	≤1.9, ≤4%/≤6.5	186	12	51/49%
Tubic	2016	Sweden	Cross-sectional	Overweight or obesity	NR	Healthy	Total and ucOC	ELISA	≤3.3%	108	2–9	NR
Khoshhal	2015	Saudi Arabia	Cross-sectional	Patients diagnosed with T1DM for 3 years or more without signs of puberty	Patients with a history of fractures of less than a year and those with associated bone/joint problems, liver disease or patients on long-term steroid theray, or incomplete data	T1D	Total OC	ELISA	NR	75	7.94	65/35%
Loureiro	2014	Brazil	Cross-sectional	Children and adolescents with T1D (ADA)	Subjects with bone diseases or increased bone loss, inflammatory and infectious diseases, history of alcohol intake, smokers or pregnant women were not included in the study	T1D	Total OC	ECLIA	NR	175	11.5	46/54%
Maggio	2012	Switzerland	Cross-sectional	Children and adolescents with T1D	Presence of other chronic diseases including thyroid or gastrointestinal diseases; medications, hormones other than insulin or calcium preparations, which may influence bone accretion taken in the preceding 6 months; presence of nephropathy; systemic disease or hospitalization for more than 2 weeks in the preceding year; and participation in competition sport, which may be more prevalent in the healthy group and further increase BMD.	T1D	Total OC	ECLIA	1.1%–5.9%	59	10.5	49/51%
Abd El Dayem	2011	Egypt	Cross-sectional	Height more than 2 SDS, diagnosis of T1DM before 18 years of age, no evidence of diabetic retinopathy, neuropathy of nephropathy; no intake medications, hormones, vitamins, or calcium in the preceding 6 months aside from insulin or thyroid hormones, no chronic disease, no hospitalization or DKA in the preceding months, no restriction of physical activity.	Height less than 2 SDS, diagnosis of T1DM after 18 years of age, evidence of diabetic retinopathy, neuropathy of nephropathy; intake medications, hormones, vitamins, or calcium in the preceding 6 months aside from insulin or thyroid hormones, no chronic disease, no hospitalization or DKA in the preceding months, no restriction of physical activity.	T1D	Total OC	EIA	3.1%–4.7%/3.5%–5.6%	77	13	33/77%
Pollock	2011	EUA	Cross-sectional	White or black/African-American race, age 7–11 years, overweight [body mass index (BMI) 85th percentile for age and sex and sedentary (no regular participation in an exercise program more than 1 h per week)	Taking medications or had any medical conditions that could affect growth, maturation, physical activity, nutritional status, or metabolism or were unable to provide blood samples	Prediabetes	Total OC, uOC	RIA	NR	140	9.2	57/43%
Maggio	2010	Switzerland	Cross-sectional	Children and adolescents with T1D	Presence of other chronic disease; medications, hormones, (other than insulin), or calcium preparations taken in the pre-ceding 6 months, presence of nephropathy, systemic disease or hospitalization for more than 2 weeks in the preceding year less than 6 menstrual cycles in the past year for post-menarchal girls	T1D	Total OC	ECLIA	1.1%–5.9%	59	10.5	49/51%
Pater	2010	Poland	Cross-sectional	Children and adolescents with T1D	Chronic diseases other than T1D and had taken medicines affecting bone metabolism	T1D	Total OC	ELISA	NR	92	11.9	68/32%
Pollock	2010	EUA	Cross-sectional	White or black/African-American race, age 7–11 years, overweight [body mass index (BMI)85th percentile for age and sex and sedentary (no regular participation in an exercise program more than 1 h per week)	Taking medications or had any medical conditions that could affect growth, maturation, physical activity, nutritional status, or metabolism or were unable to provide blood samples	Prediabetes	Total OC, ucOC	RIA	NR	140	9.1	57/43%
Prats-Puig	2010	Spain	Cross-sectional	Age between 5–9 years and absence of puberty	Evidence of acute or chronic illness	Healthy	Total and ucOC	EIA	NR	103	5–9	47/53%
Karagüzel	2006	Turkey	Cross-sectional	Children and adolescents with T1D (ADA criteria)	NR	T1D	Total OC	ECLIA	NR	102	11.2	49/51%
Ersoy	1999	Turkey	Cross-sectional	T1D, age ≤21 years, no disease known to affect bone metabolism, no chronic disease, weight and height between 10th or above 97th centile	Diseases known to affect bone metabolism, weight or height score below 10th or above 97th centile, any chronic disease	T1D	Total OC	NR	NR	53	11–16	50/50%
Gunczler	1998	Venezuela	Cross-sectional	Children with T1D and no other chronic disease, height and weight between 5th–90th centile	Any chronic disease other than diabetes was present	T1D	Total OC	RIA	3.6%–4.4%/3.1%–5.8%	53	7.1–14.3	56/44%
Leon	1989	Spain	Cross-sectional	Children and adolescents with T1D	NR	T1D	Total OC	RIA	NR	136	9.7	58/42%

The Newcastle–Ottawa scale was used for the quality assessment of observational studies. All studies were considered as high quality (had 6 or more points) (10, 11, 15–17, 30–35, 38, 39, 41–44) (Table 2).

**Table 2:** Quality assessment of the studies included with the the Newcastle–Ottawa scale.

Study		Selection			Comparability			Exposure		Stars
Author	Year	Is the case definition adecuate?	Representativeness of the cases	Definition of controls	Study controls for main outcome	Study controls for additional outcomes	Ascertainment of exposure	Same method of ascertainment for cases and controls	Non-Response rate	
Abd El Dayem	2011	*	*	*	*	*	*	*	*	8
Ersoy	1999	*	*	*	*	–	*	*	*	7
Faienza	2016	*	*	*	*	–	*	*	*	7
Faienza	2018	*	*	*	*	–	*	*	*	7
Giudici	2017	*	*	*	*	*	*	*	*	8
Gunczler	1998	*	*	*	*	*	*	*	*	8
Huneif	2017	*	*	*	*	*	*	*	*	8
Karagüzel	2006	*	*	*	*	*	*	*	*	8
Khoshhal	2015	*	*	*	*	*	*	*	*	8
Leon	1989	*	*	*	*	*	*	*	*	8
Loureiro	2014	*	*	*	*	*	*	*	*	8
Maggio	2010	*	*	*	*	–	*	*	*	7
Maggio	2012	*	*	*	*	–	*	*	*	7
Pater	2010	*	*	*	*	*	*	*	*	8
Takaya	2017	*	*	*	*	*	*	*	*	8
Pollock	2010	*	*	–	–	*	*	*	*	6
Pollock	2011	*	*	–	–	*	*	*	*	6
Tubic	2016	*	*	*	*	*	*	*	*	8
Prats-Puig	2010	*	*	*	*	*	*	*	*	8
Redondo	2017	*	*	*	*	*	*	*	*	8

*means the paper fulfill such quality criteria.

From the 20 included studies, a total of 3,000 pediatric patients underwent tOC, cOC, or ucOC assessment, including 983 with T1D, 30 with T2D, and 1987 healthy individuals. A total of 14 studies were performed in T1D (10, 15, 16, 30, 32, 34, 35, 39–45), two in T2D (11, 45), two in prediabetes, and three in HS. The mean patient age at inclusion was 10.6 years, and the male-to-female ratio was 1 : 0.9. The methods used for osteocalcin measurement were EIA in 5 studies (30, 35, 38, 45, 46), RIA in 4 studies (36, 37, 40, 41), ELISA in 6 studies (11, 29, 31, 43), ECLIA in 4 studies (16, 34, 42) and in one study the technique for quantification of osteocalcin was not reported. The intra-and interassay coefficients of variation were from 1.1% to 10.8%.

### Comparisons of total OC between T1D and HS

**Figure 2:**
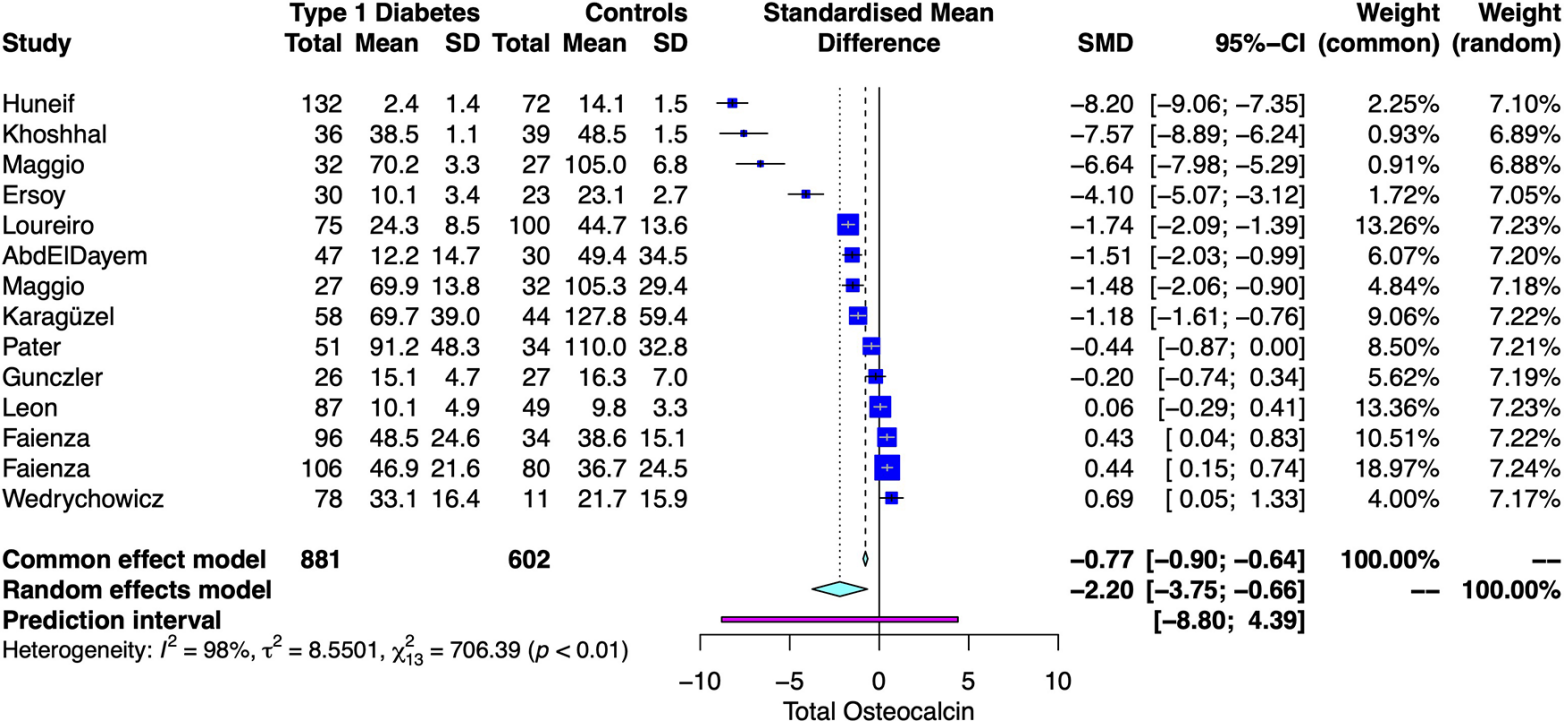
Forest plot with the individual results and the pooled estimates of tOC serum levels among T1D and controls.

A total 14 studies compared the total OC serum concentrations among T1D and HS (15–17, 30, 33–35, 39–43, 47). There was lower SMD for serum levels of total OC in T1D than in HS (SMD: −2.2, CI 95%: −3.75 to −0.66). A *Q* value of 706.39 with 13 degrees of freedom and *p* < 0.001 provides evidence that the effect size varies across studies, and *I*^2^ indicates that 98.0% of the variation can be attributed to a true effect rather than a random error (Figure 2).

**Figure 3:**
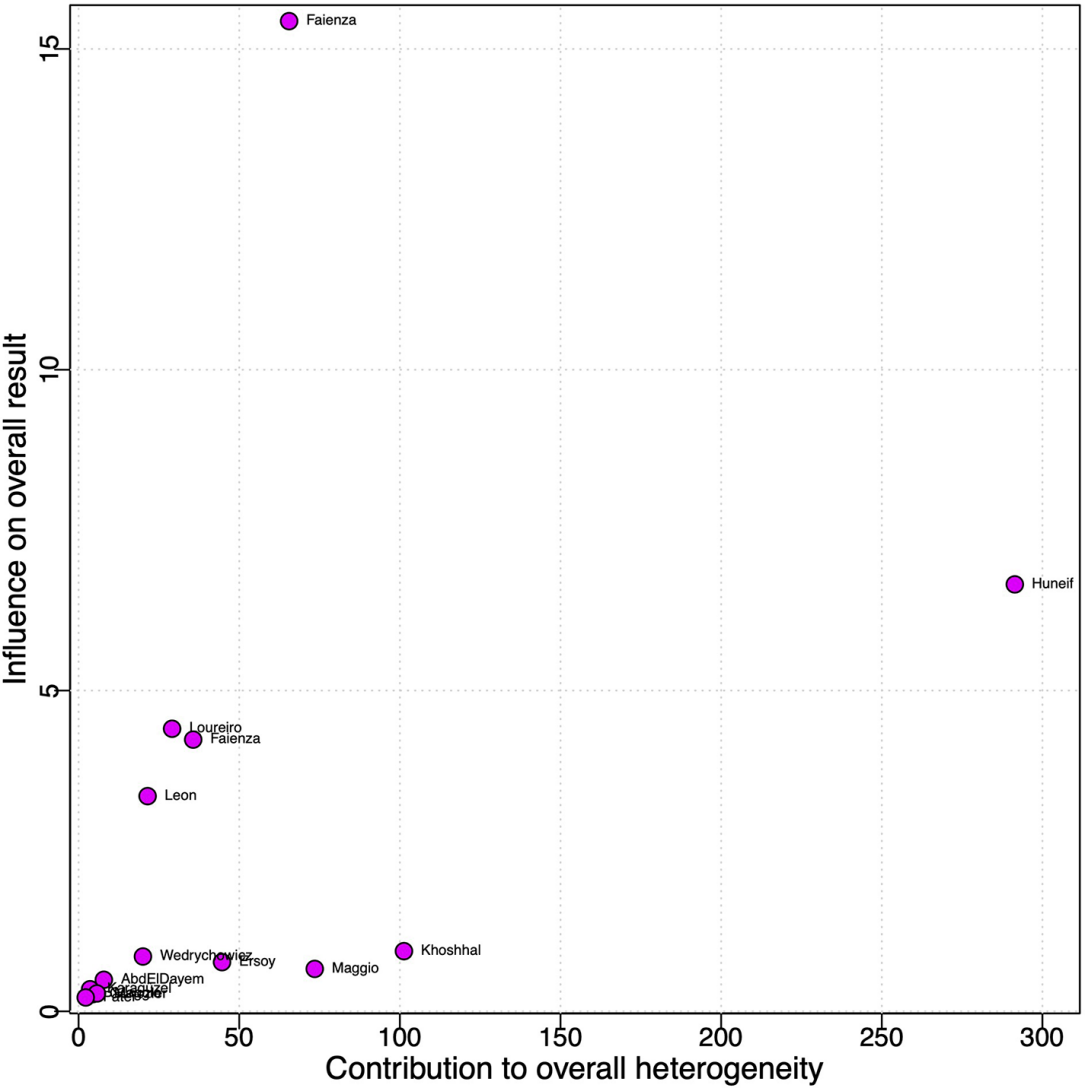
Baujat plot for analysis of the contribution of each study to heterogeneity in tOC serum levels.

Baujat plot showed that the studies from Faienza influenced the most to the overall results, whereas Huneif contributed the most to the overall heterogeneity (Figure 3) (15–17, 30, 35, 39, 41). The funnel plot suggested publication bias (Supplementary S2). The cumulative analysis showed no “small-study effect” on effect size (Supplementary S3).

### Comparison of ucOC in abnormal glycemic status vs. HS

**Figure 4:**
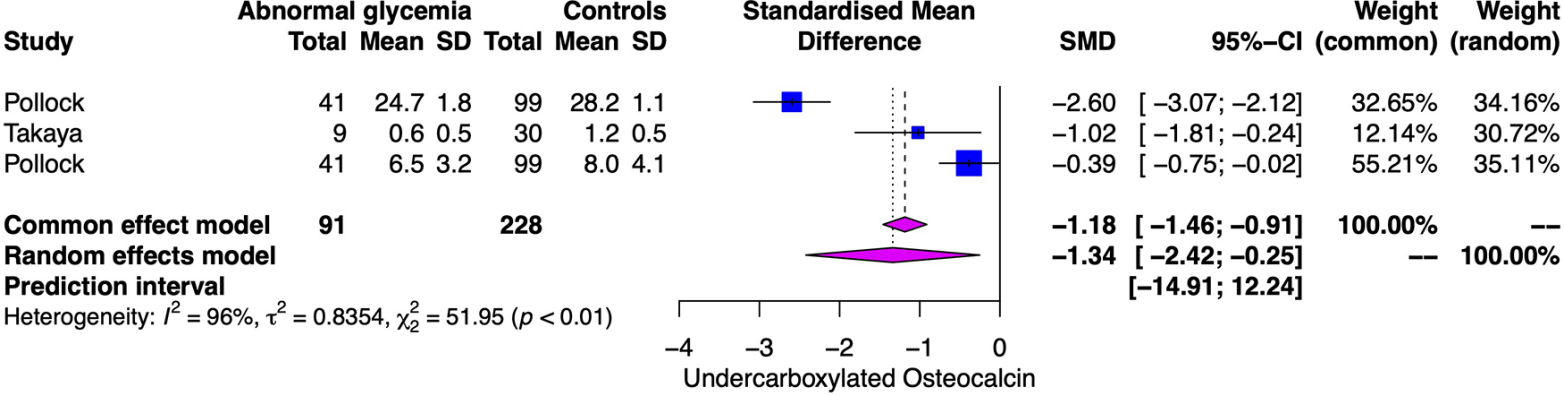
Forest plot with the individual results and the pooled estimates of ucOC serum levels among patients with abnormal glycemic status and controls.

In total, three studies compared the ucOC serum levels among individuals with abnormal glycemic status (AGS* *= T2D or prediabetes) and normal glycemic status. There was lower SMD for serum levels of ucOC in AGS than in HS (−1.34, CI95%: −2.42 to −0.25). A *Q* value of 51.95 with 2 degrees of freedom and *p* < 0.001 provides evidence that the effect size varies across studies, and *I*^2^ indicates that 96% of the variation can be attributed to a true effect rather than a random error (Figure 4) (11, 36, 37).

### Comparison of ucOC between T1D and HS

Only Huneiff measured ucOC in T1D and found lower serum levels in these patients (1.08 ± 0.56 ng/ml) than in HS (2.58 ± 0.55 ng/ml) (17).

### Comparison of ucOC between T2D and HS

One study measured ucOC serum concentrations in pediatric patients with T2D, reporting lower ucOC serum levels among T2D (0.65 ± 0.46 ng/ml) than in controls (1.25 ± 0.49 ng/ml; *p* < 0.01) (11).

### Correlation of ucOC with metabolic parameters in HS and abnormal glucose parameters

**Figure 5:**
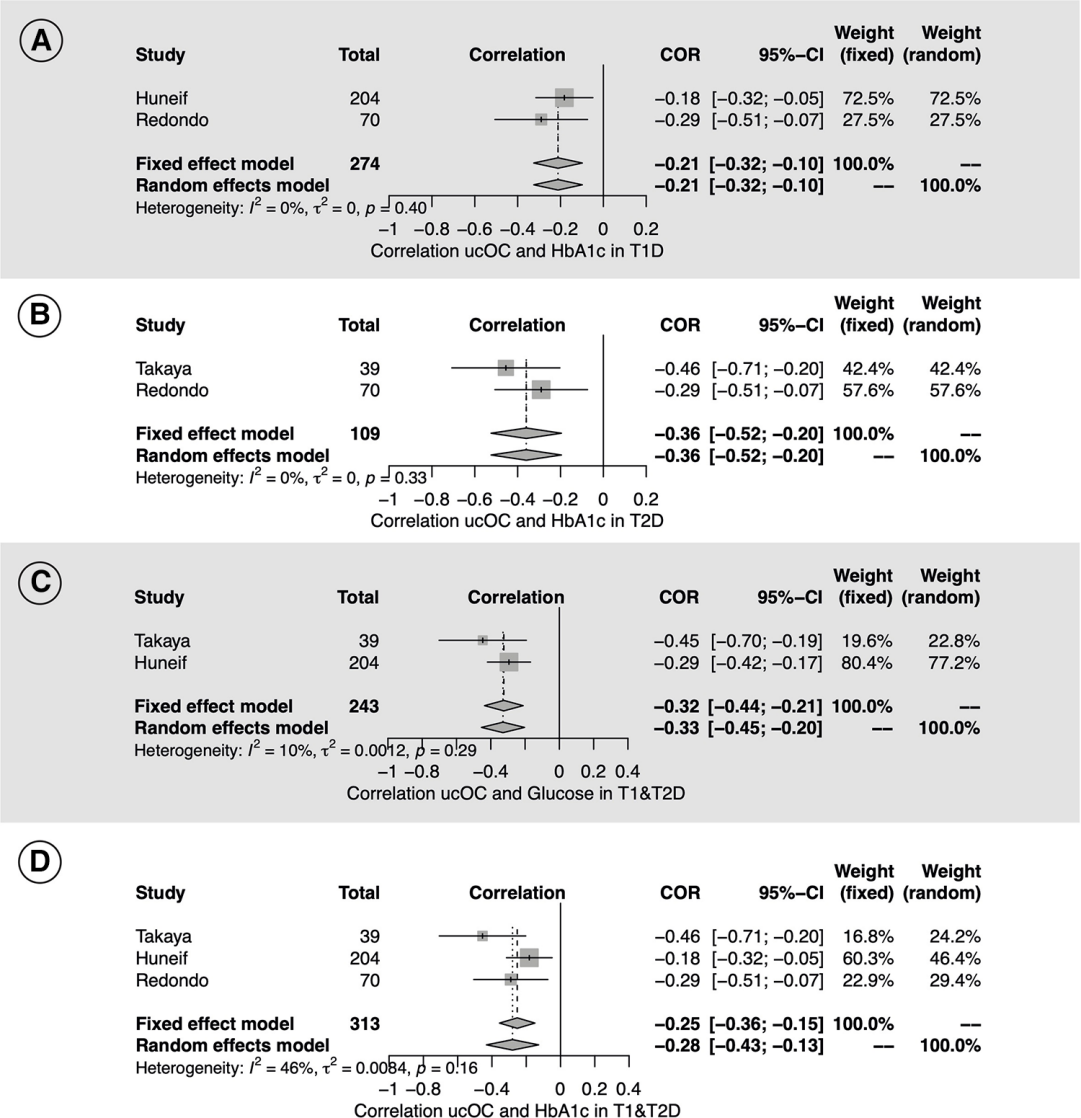
Forest plot with the individual results and the pooled estimates of the Pearson correlation coefficients (COR) of ucOC serum levels with HbA1c and glycemia among T1D and T2D pediatric patients.

A total of two studies reported correlations of ucOC with metabolic and anthropometric parameters in HS (29, 31) and three in diabetic patients (11, 17, 45). In healthy subjects, significant positive correlations of ucOC with WC and weight were found but not with other parameters (Supplementary S4). In diabetic children, significant negative correlations of ucOC with HbA1c and glycemia were found in T1D and T2D (Figure 5).

### Correlation of tOC with anthropometric and metabolic parameters in HS and diabetes

Two studies reported correlations of tOC with metabolic and anthropometric parameters in HS (29, 31) and two in diabetic patients (30, 44). In HS, a significant positive correlation was identified for FPG, LDL-c, WC, height, and weight (Supplementary S5A–J). Among T1D children, a significant negative correlation was also found between tOC and HbA1c, but no data regarding T2D were identified in publications (Supplementary S5K).

### cOC

One report compared the serum levels of cOC between HS and T2D and found no significant differences (11). No differences in T1D or T2D. Only one study performed in healthy children found that high serum levels of cOC were related to lower HMW-adiponectin in leaner children (a hormone released by osteocalcin in animals that increases insulin sensitivity). Moreover, they found that a higher ucOC-to-cOC ratio was associated with higher HOMA-β in leaner children and related with higher HMW-adiponectin in heavier children, concluding that carboxylation of osteocalcin relates to metabolic and anthropometric parameters in a weight-dependent manner (38).

### Contributors to the variability of tOC

**Figure 6:**
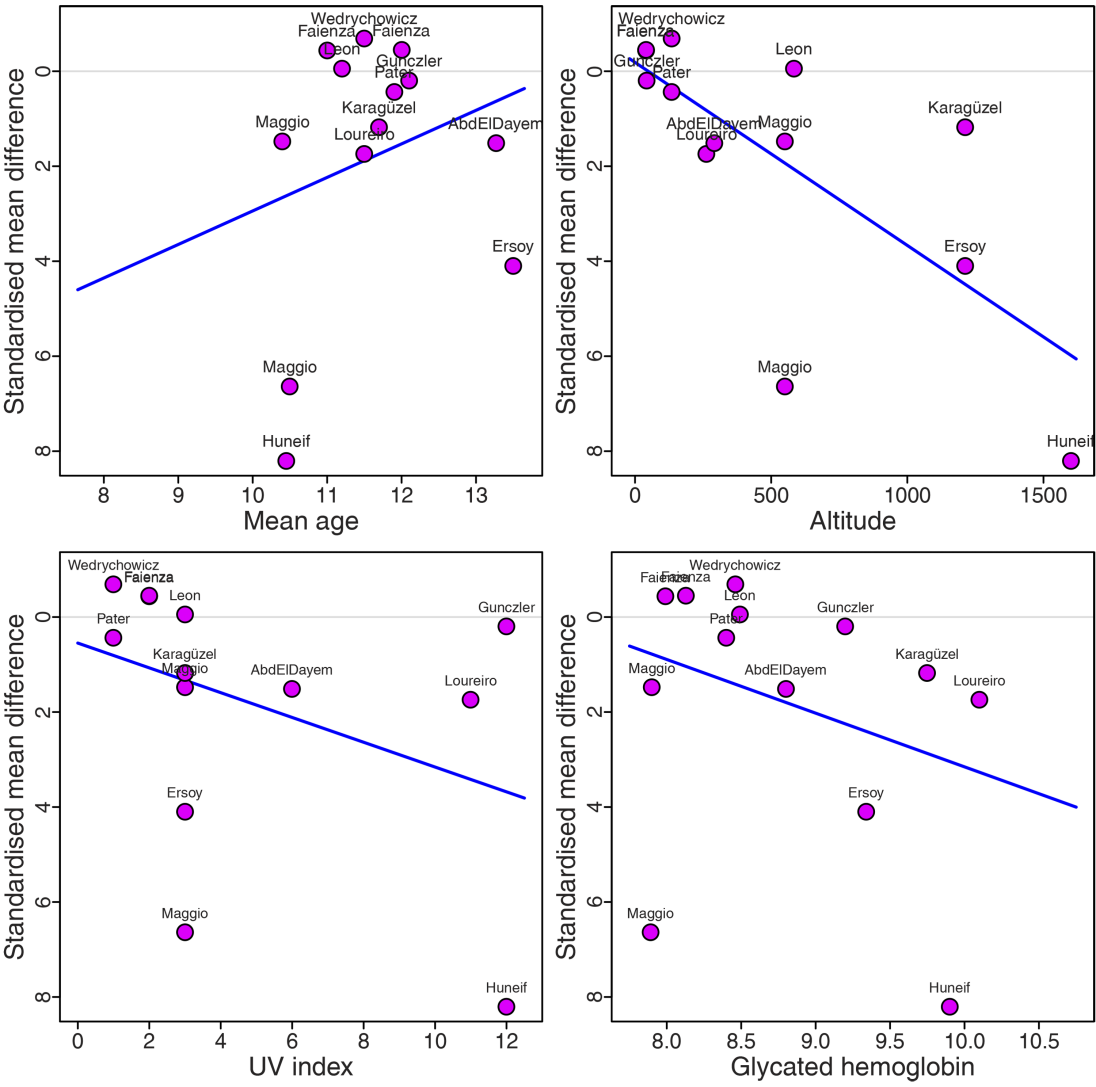
Meta-regressions showing variables influencing the serum levels of tOC in pediatric patients: (**A**) mean age. (**B**) Altitude. (**C**) UV index. (**D**) Glycated hemoglobin.

Since variability was found to be high for tOC estimates, univariate and multivariate meta-regressions were performed to determine which variables influence the serum levels of tOC. In the individual meta-regressions, covariates significantly explaining the heterogeneity were patient age (*R*^2 ^= 25.53%), altitude (*R*^2 ^= 34.67%) and HbA1c (*R*^2 ^= 26.43%) (Table 3; Figure 6).

**Table 3:** Univariate meta-regression analysis of intrinsic and extrinsic determinants for tOC in T1D.

Covariat3	*Tau* ^2^	*tau*	*I*^2^ (%)	*H* ^2^	*R*^2^ (%)	Estimate	95% CI		*p* value
**Age**	**6**.**3673**	**2**.**5234**	**99**.**05**	**105**.**18**	**25**.**53**	**1**.**1497**	**0**.**1157**	**2**.**1838**	**0**.**029**
**Altitude**	**5**.**5861**	**2**.**3635**	**98**.**88**	**89**.**13**	**34**.**67**	**−0**.**0035**	**−0**.**0061**	**−0**.**0009**	**0**.**008**
UV index	7.2720	2.6967	99.12	113.36	14.95	−0.2899	−0.0661	0.0812	0.126
**Glycated hemoglobin**	**6**.**2899**	**2**.**5080**	**98**.**97**	**97**.**05**	**26**.**43**	**−1**.**7078**	**−3**.**2366**	**−0**.**1790**	**0**.**0286**

The bold values indicate significant covariates.

The multivariate meta-regression indicates that 62.8% of the total heterogeneity was explained by the covariate age, altitude, and HbA1c. In the moderator test, the estimated model was highly significant with QM (*df* = 4) = 41.21 and *p* < 0.0001. The patient age (*p* = 0.007), altitude (*p* = 0.008), and HbA1c (*p* = 0.012) have a significant influence on the levels of tOC serum, while the UV index was not significant (*p* = 0.64) (Table 4).

**Table 4:** Multivariate meta-regression analysis of intrinsic and extrinsic determinants for tOC in T1D^a^.

Covariate	Estimate	95% CI		*p* value	R2 (%)^a^
**Age**	**0**.**7557**	**0**.**2065**	**1**.**3049**	**0**.**007**	62.8
**Altitude**	**−0**.**0028**	**−0**.**0048**	**−0**.**007**	**0**.**008**	
UV index	−0.0682	−0.3547	0.2182	0.640	
**Glycated hemoglobin**	**−1**.**0108**	**−1**.**8015**	**−0**.**220**	**0**.**012**	

^a^ **Other estimates:** *Tau*^2^*^ ^*=*^ ^*3.1837, *tau* = 1.7843, H^2 ^= 50.

The bold values indicate significant covariates.

The estimated model indicates that every one-year increase in the patient's age corresponds to an increment of 0.7557 units in terms of the SMD, while keeping the other covariates constant. Similarly, for every unit of altitude increasing, the true effect size decreases at a rate of 0.0028. Finally, for every one-unit change in HbA1c, the SMD of levels of tOC serum decreases at a rate of 1.01.

Residual analysis was performed (see Supplementary S6), in which it can be noted that the assumptions of homoscedasticity, independence, and normality are satisfied—such analysis allowed the validation of the meta-regression model.

## Discussion

### Main findings

This meta-analysis revealed the following findings:

(i) *Among healthy children there was a:*
    (a) Positive correlation of ucOC with WC and weight
    (b) Positive correlation between tOC and the following parameters: FPG, LDL-c, WC, height, and weight; and a negative correlation between tOC and HbA1c.
(ii) *Among diabetic children there was a:*
    (a) Negative correlation of ucOC with HbA1c and glycemia in both T1D and T2D.
    (b) Negative correlation between tOC and HbA1c in T1D but not in T2D.
(iii) *In the comparison of diabetic* vs. *healthy subjects:*
    - ucOC concentrations were lower in T2D, T1D, and patients with abnormal glucose status than among controls.
    - Serum concentrations of tOC concentrations were lower in T1D than in controls.
(iv) *The factors influencing serum* *levels of tOC* were age (+), altitude (−), and glycated hemoglobin (−) explaining together 62.8% of the heterogeneity.

### Clinical implications and physiological plausibility of the findings

Since obesity, insulin resistance, glycemic, and lipid abnormalities predispose to children and adolescents to the development of chronic diseases in adulthood such as diabetes, hypertension, and cardiovascular diseases, it is quite important to identify serum markers related to abnormal metabolic phenotypes. Because osteocalcin was consistently related to FPG, HbA1c, LDL-c, WC, height, and weight in infants and adolescents, its routine clinical measurement as a marker children/adolescent metabolic status is plausible. Especially because ucOC has several metabolic effects discovered in preclinical studies, such as increased insulin synthesis, beta-cell proliferation, and insulin sensitivity throughout an action on the pancreas, adipose, and muscle tissue (48). Furthermore, a decrease in ucOC is related to fat accumulation in adipocytes and hepatocytes (49). In contrast, the administration of this hormone decreases the content of triglycerides at these cells (50), improves glucose metabolism, and prevents T2D in mice. Thus, a plausible biological explanation exists for our findings about the correlation of ucOC with glucometabolic and anthropometric parameters.

### Strengths and limitations

Our study has several strengths. Firstly, we conducted a rigorous systematic review by independent reviewers and a third one to evaluate and assess bias. Database searching was also done by independent investigators, all blinded to authorship and the hospital where the study was conducted, allowing us to minimize bias when selecting publications for inclusion. Secondly, the standardized mean difference was used to avoid discrepancies in unit measurements along with an exhaustive assessment of heterogeneity by 10^6^ simulations, subgroup analysis, and meta-regression. In addition, multiple methods were used to measure bias in each group, from advanced techniques such as the Copas model to cumulative analysis, aiming to determine the trend toward higher effects in smaller studies. Finally, multiple intrinsic and extrinsic factors were analyzed by single and multivariate meta-regression to address the influence of all these factors on the variability of tOC. It allows us to understand that tOC levels were mainly influenced by age, glycemic control, and altitude but not by latitude, BMI, insulin, or other covariates. This complete analysis is a strength because it should be considered when designing clinical research involving osteocalcin or stablishing reference values. On another way, we must mention some weaknesses, including a relatively small number of studies performed in children with T2D, the use of different methods (RIA, EIA, ELISA) to measure the serum levels of osteocalcin and the non-reporting of the inter-assay coefficients of variation for all the studies. In addition, we cannot assure that the primary studies considered all factors that modify OC serum levels such as the treatment, exercise, and the season when osteocalcin was measured.

### Conclusions and implications

Osteocalcin is involved in energy metabolism in pediatric subjects because it is consistently related to FPG, HbA1c, LDL-c, and anthropometric parameters such as WC, height, and weight. Although serum levels of OC are highly variable among populations, such variability is explained by diverse intrinsic and extrinsic factors such as age, altitude, and glycated hemoglobin.

Moreover, its utility as a predictor of metabolic disease risk should be explored in the future, together with potential strategies to increase uCOC serum levels to improve metabolic status.

## Condensation

This meta-analysis provides evidence that osteocalcin is related to markers of energetic metabolism among pediatric patients with normal and abnormal glycemic status.

## Data Availability

The original contributions presented in the study are included in the article/**Supplementary Material**, further inquiries can be directed to the corresponding author.
