# Supplementary material for: Osteocalcin serum concentrations and markers of energetic metabolism in pediatric patients. Systematic review and metanalysis
Source: Front Pediatr. 2023 Jan 12;10:1075738. doi: 10.3389/fped.2022.1075738 (PMC9878130; doi:10.3389/fped.2022.1075738)
Supplement: Supplementary file 1 [file Image1.pdf]

SUPPLEMENT 1A. QUERY KEYWORDS

**PubMed Query**<br>(("osteocalcin"[MeSH Terms] OR "osteocalcin"[All Fields]) AND ("diabetes mellitus"[MeSH Terms] OR ("diabetes"[All Fields] AND "mellitus"[All Fields]) OR "diabetes mellitus"[All Fields] OR "diabetes"[All Fields] OR ("diabetes"[All Fields] AND "insipidus"[All Fields])) NOT ("review"[Publication Type] OR "review literature as topic"[MeSH Terms] OR "review"[All Fields]) AND ("humans"[MeSH Terms] AND (English[lang] OR Spanish[lang]))
**SCOPUS Query**<br>TITLE-ABS-KEY(osteocalcin AND diabetes AND HUMAN) AND (LIMIT-TO (DOCTYPE,"ar ")) AND (LIMIT-TO (LANGUAGE,"English ") OR LIMIT-TO (LANGUAGE,"Spanish "))
**WOS search**<br>TS=osteocalcin OR, osteocalcin AND, diabetes mellitus OR, diabetes AND, mellitus OR, diabetes mellitus OR, diabetes OR, diabetes<br>**Refined by: Document type:** (ARTICLE) AND [excluding] **Data base:** (MEDLINE)<br>**Period of search:** All years.<br>Language=Auto

SUPPLEMENT 1B. REASONS OF EXCLUSION

Author	Year	Reason of exclusion
Boucher-Berry	2012	No control group included
Bouillon	1995	Mix population (adults and children)
Brandao	2007	No report of OC in control group
De Schepper	1997	No control group included
Gogas Yavuz	2011	Mix population (adults and children)
Gonzalez-Garcia	2015	No children
Ibañez	2010	It is an experimental study
Kemink	2000	No children
Kocabas	2010	Do not report OC levels
Liu	2003	Mix population (adults and children)
Mastrandrea	2008	Mix population (adults and children)
Napoli	2013	It is an experimental study
Olmos	1994	No children included
Prats-Puig	2011	No correlations; no comparisons of OC levels among diabetes and HS

Simm	2011	It is an experimental study
Thrailkill	2011	Mix population (adults and children)
Tsentidis	2016	No serum levels of OC reported; nor correlations
Verroti	1999	Mix population (adults and children)
Gajewska	2017	No diabetics; no correlations of OC with metabolic parameters; no OC serum levels
Poomthavorn	2013	No diabetics; no correlations of OC with metabolic parameters; no OC serum levels
Saggese	1991	It is an experimental study
Topaglu	2005	It was made in diabetic ketoacidosis patients (acute illness)
Diniz-Santos	2007	It reports only cases of patients with T1D with and without celiac disease
Flemming	2012	Because the study was performed only in healthy children and adolescents. It does not include diabetic patients
Tsentidis	2015	Because the correlation reported was between logOC and glycemia but not between absolute OC concentrations and glycemia
