# Supplementary figures and images for: Osteocalcin serum concentrations and markers of energetic metabolism in pediatric patients. Systematic review and metanalysis
Source: Front Pediatr. 2023 Jan 12;10:1075738. doi: 10.3389/fped.2022.1075738 (PMC9878130; doi:10.3389/fped.2022.1075738)

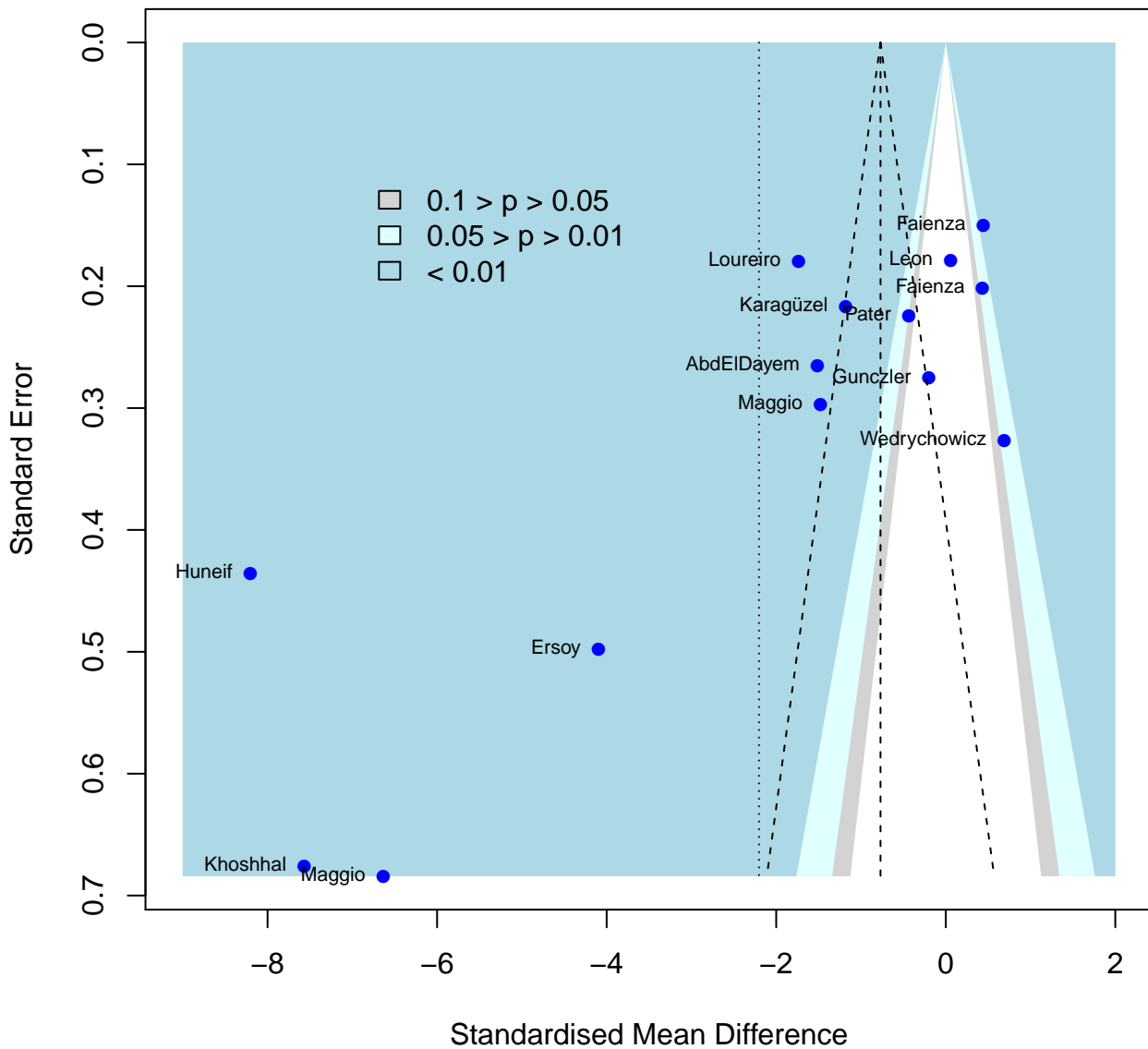

Supplement: Supplementary file 2 [file Image2.pdf]

# Total Osteocalcin

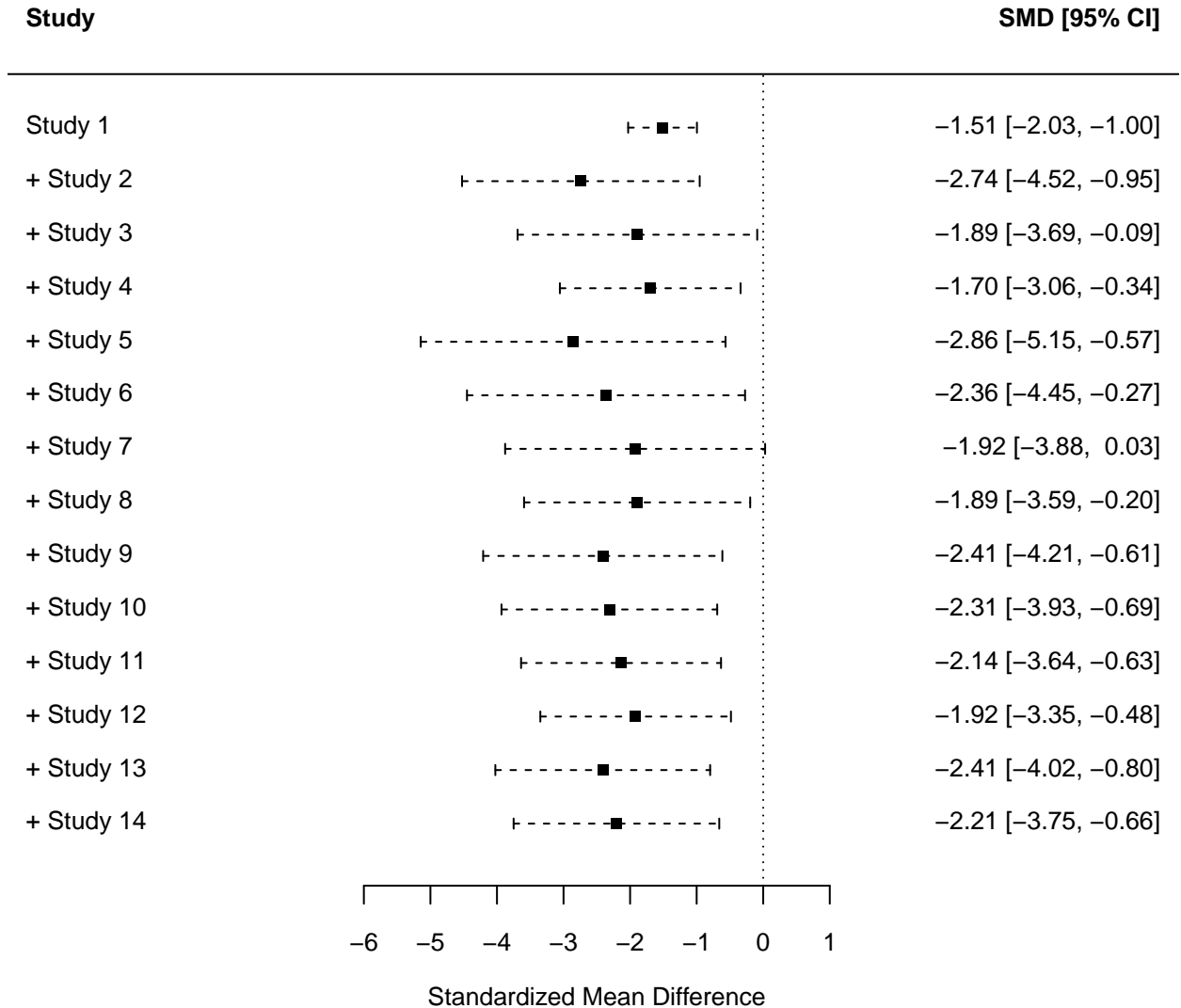

Supplement: Supplementary file 3 [file Image3.pdf]

A

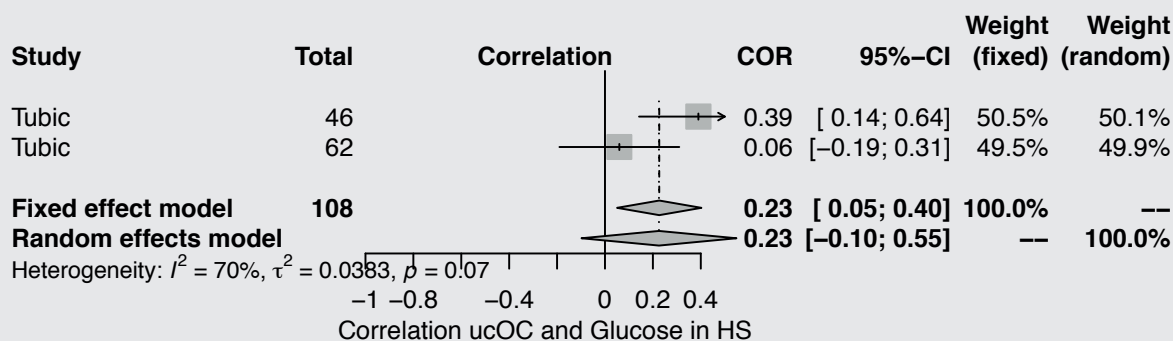

B

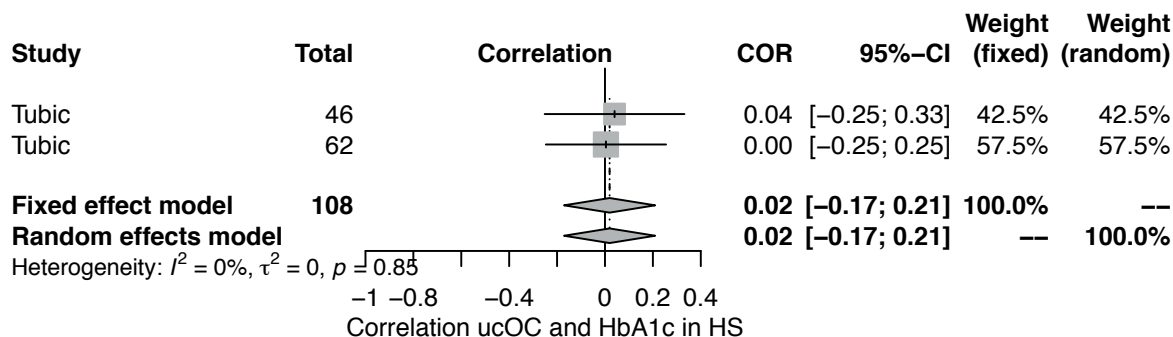

C

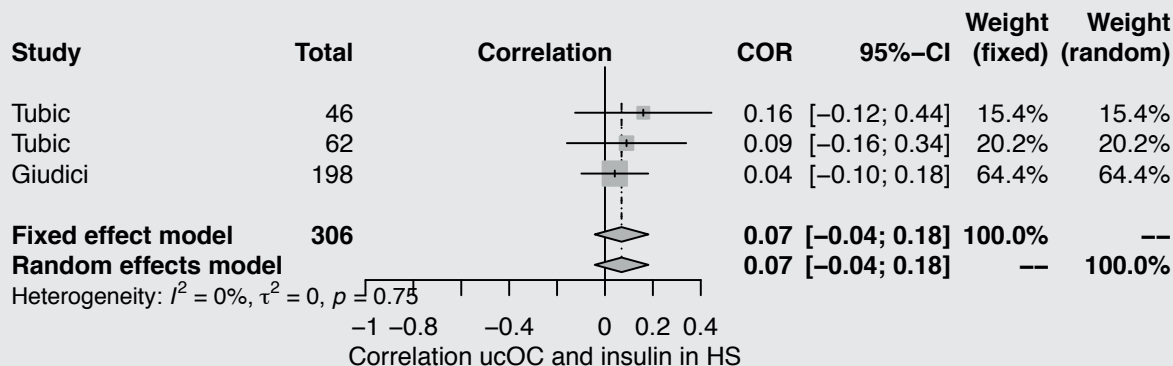

D

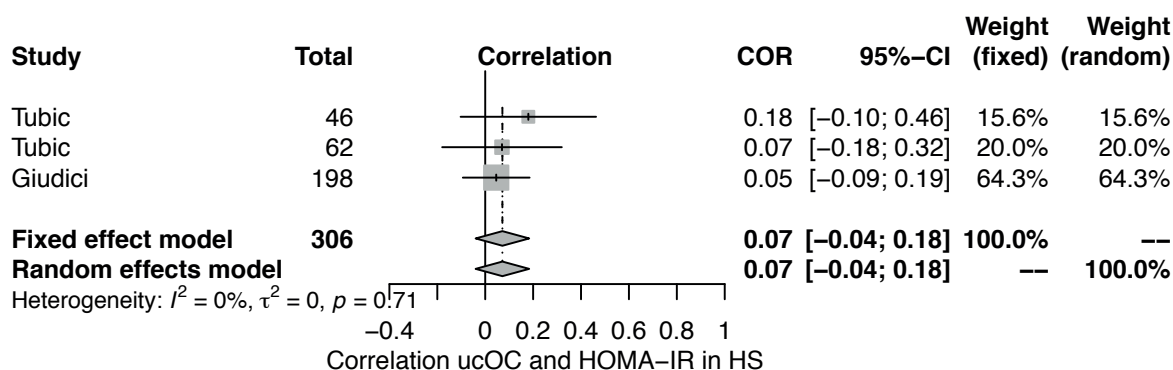

E

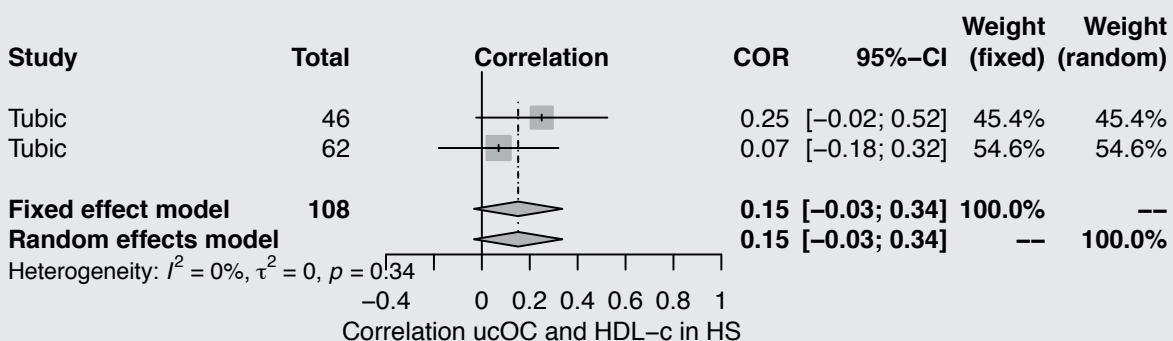

F

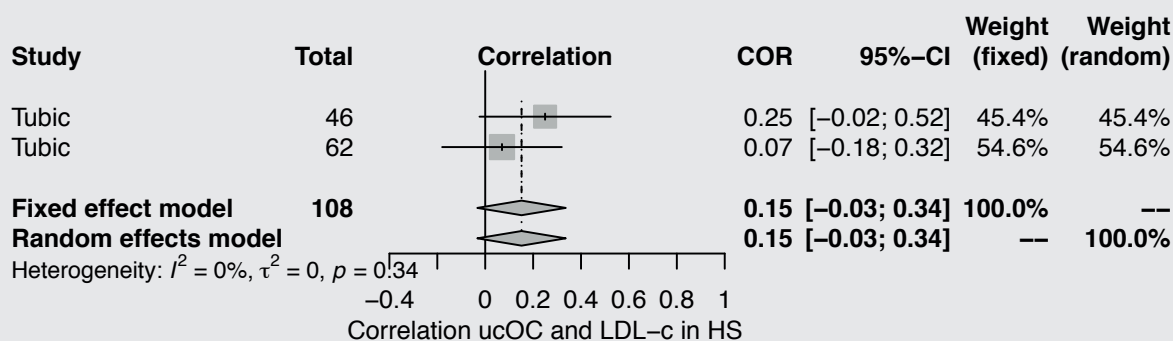

G

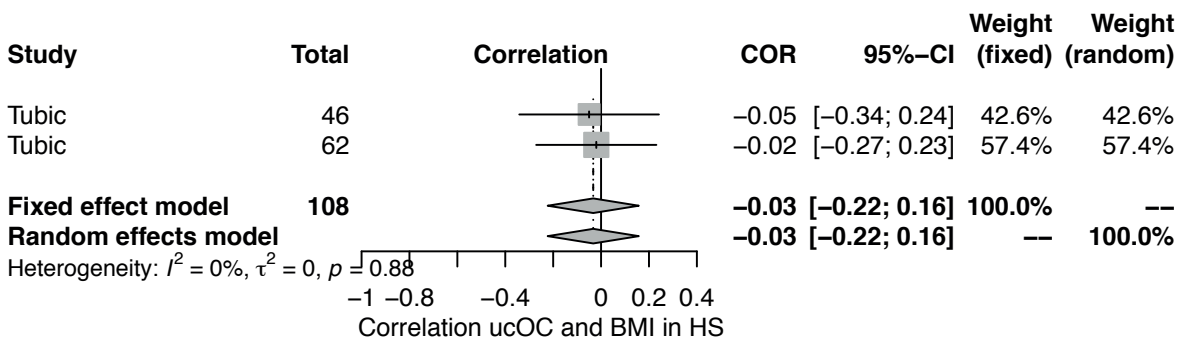

H

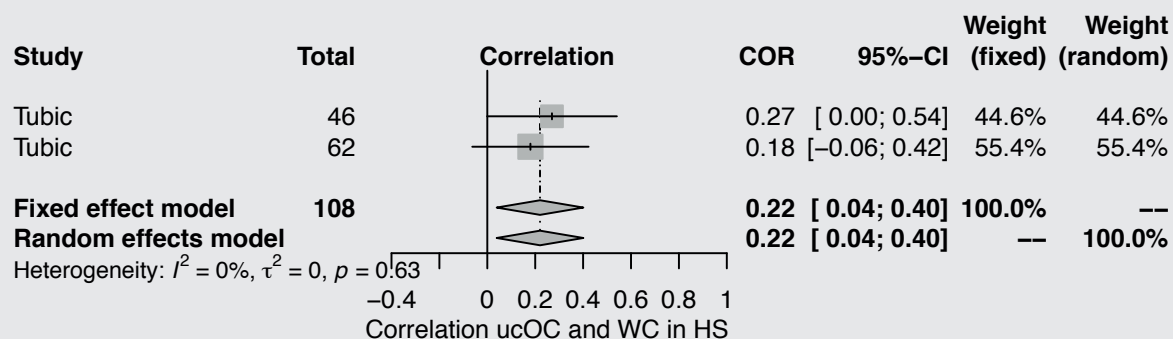

I

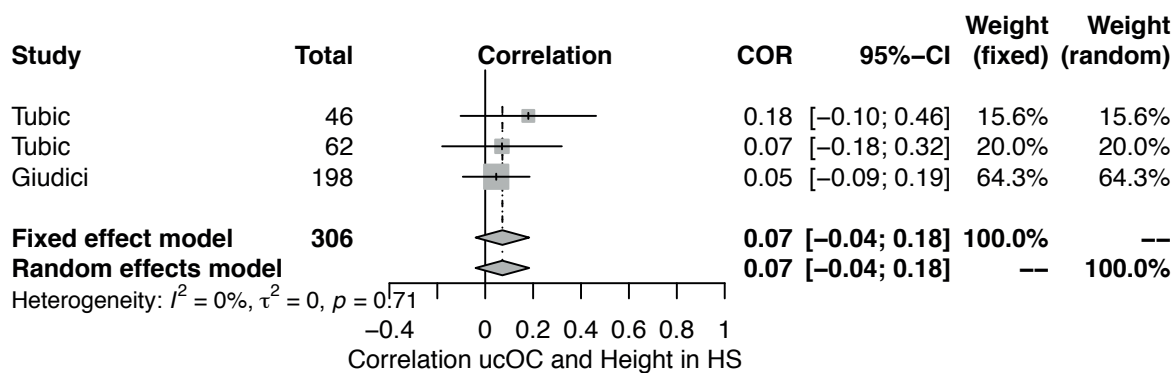

J

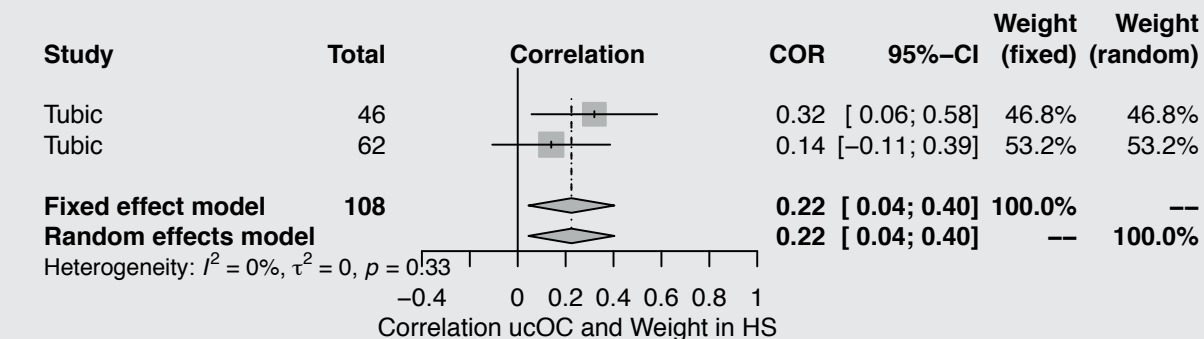

K

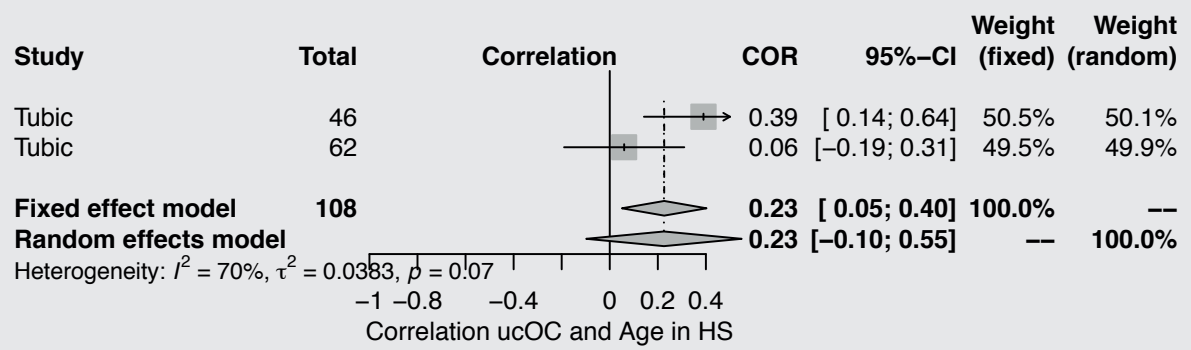

Supplement: Supplementary file 4 [file Image4.pdf]

A

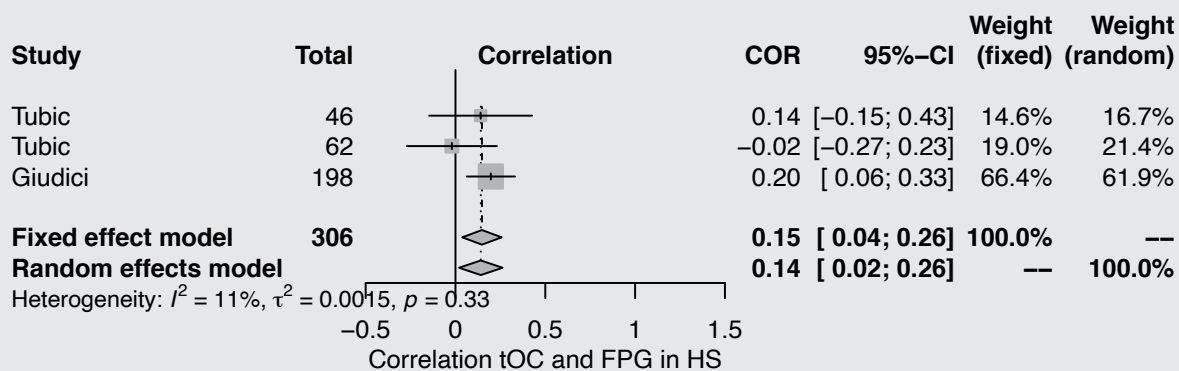

B

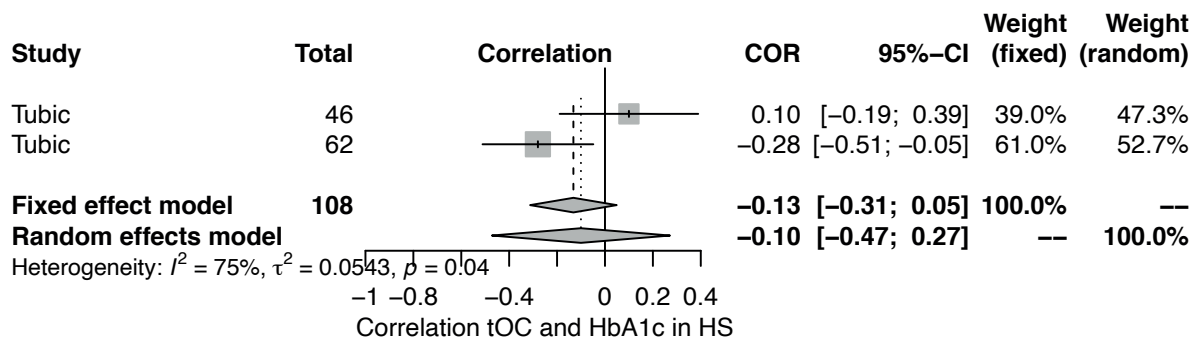

C

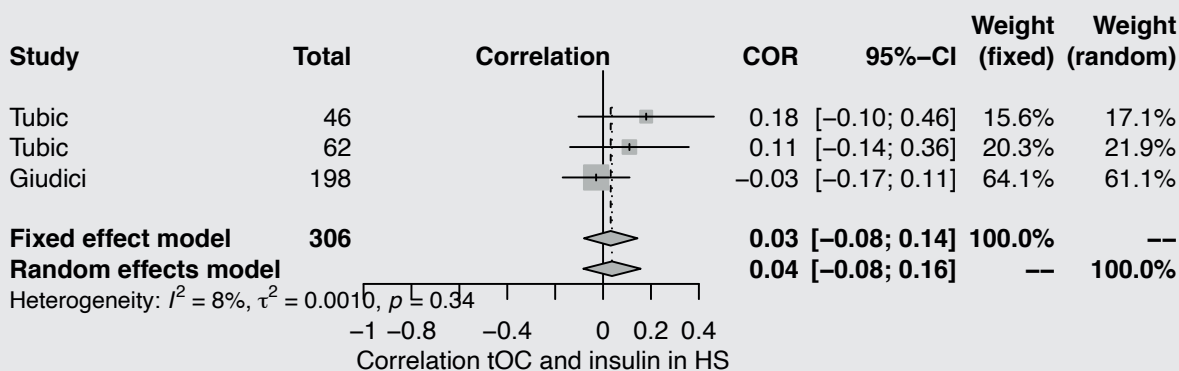

D

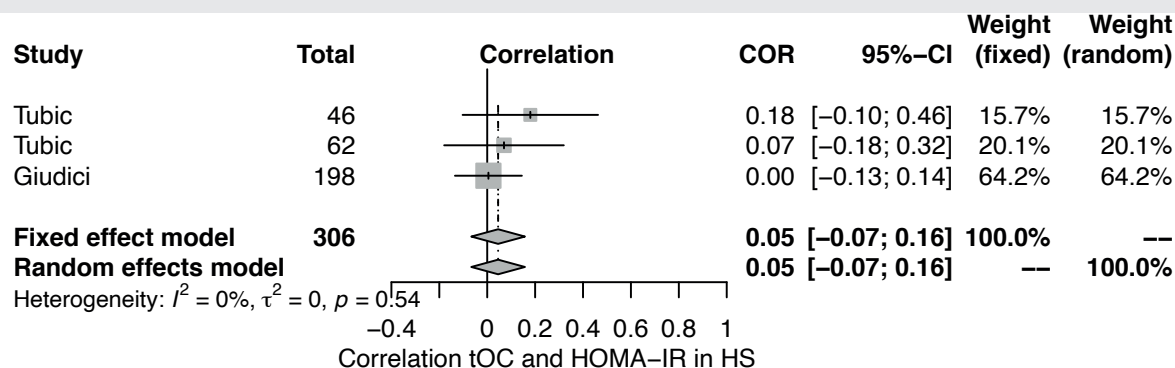

E

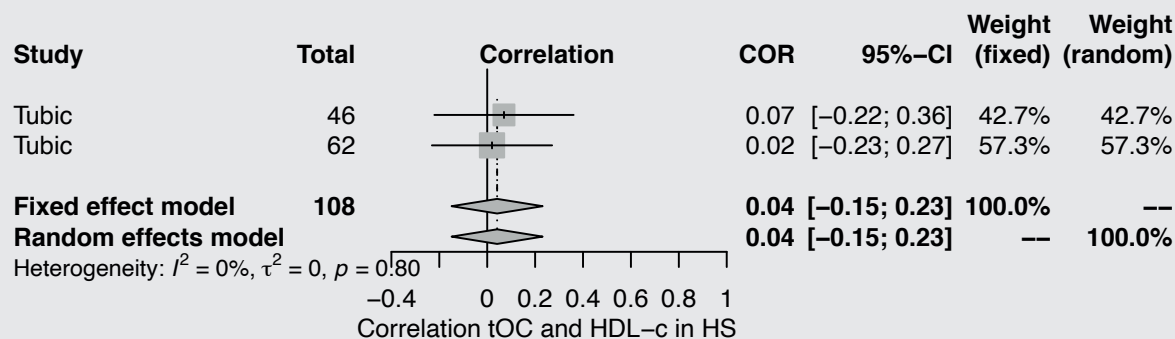

F

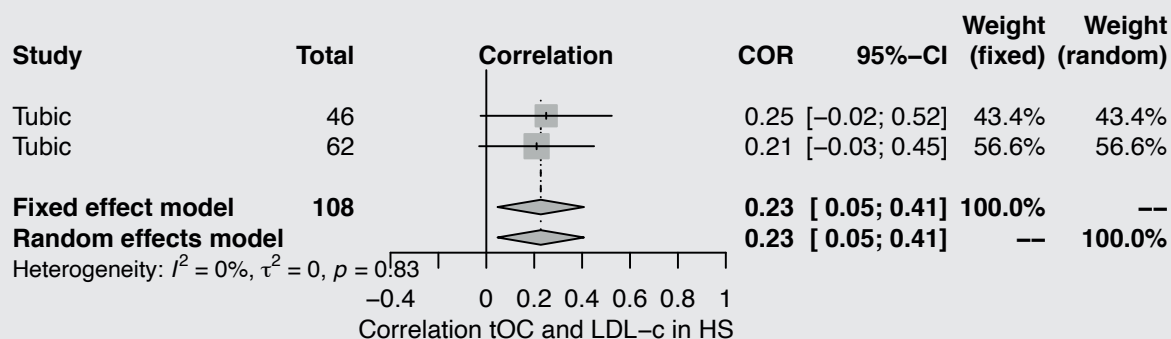

G

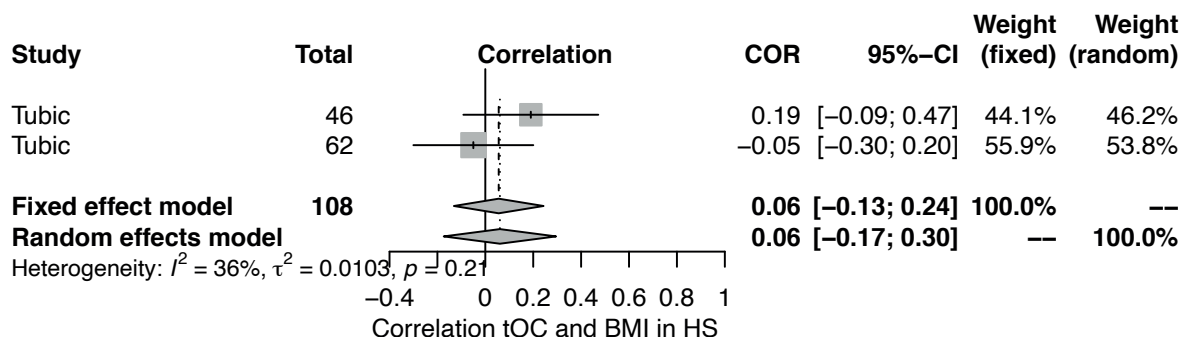

H

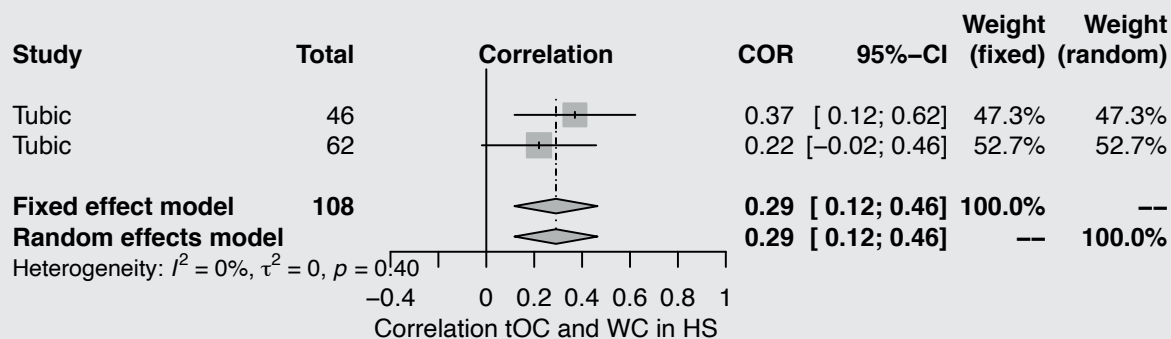

I

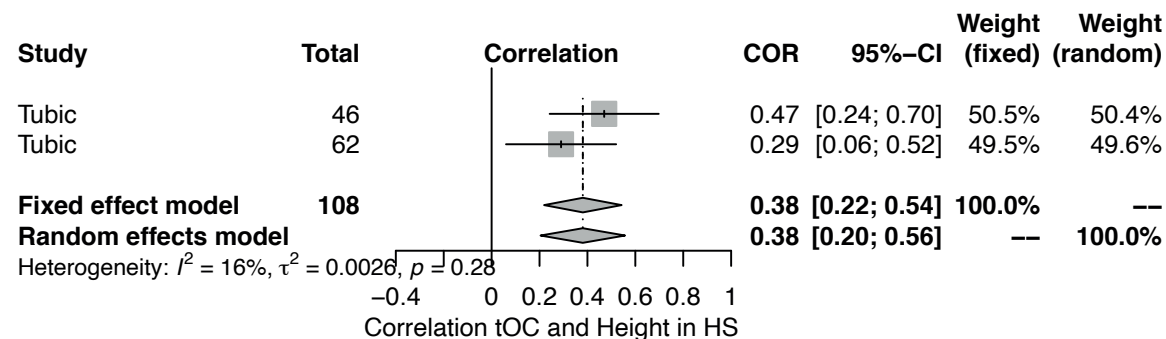

J

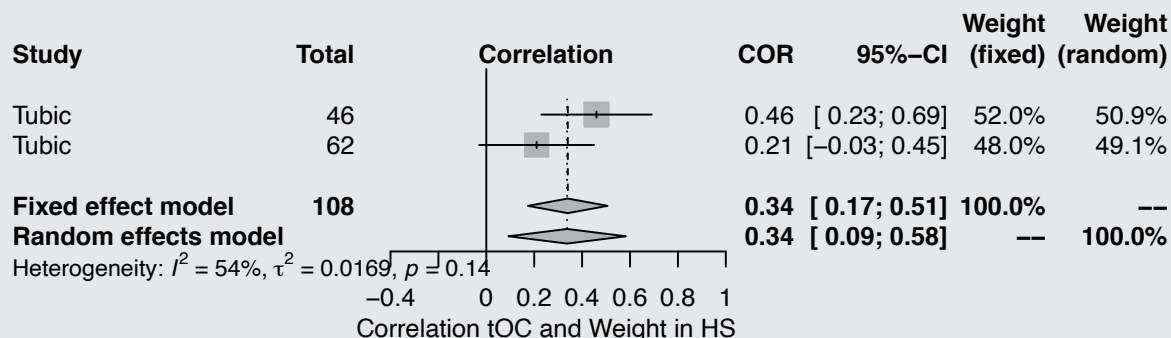

K

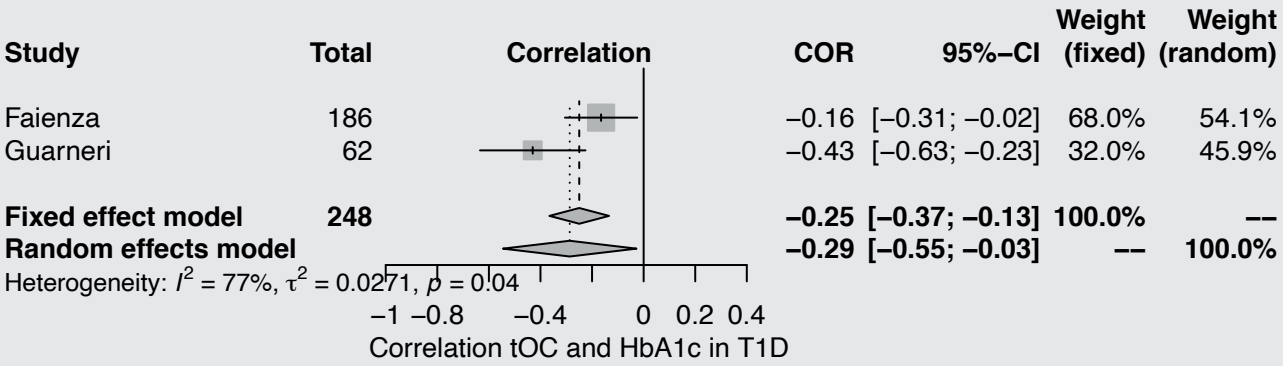

Supplement: Supplementary file 5 [file Image5.pdf]

Forest Plot

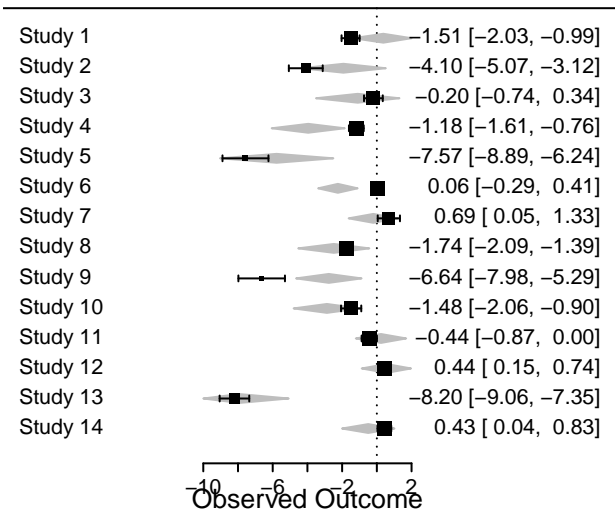

Residual Funnel Plot

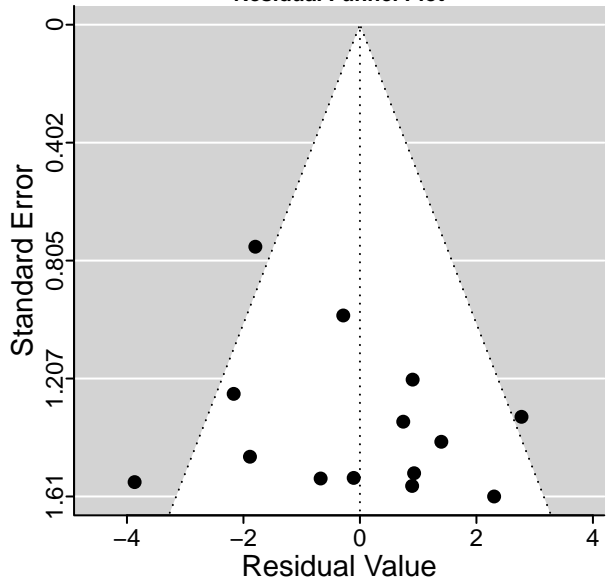

Fitted vs. Standardized Residuals

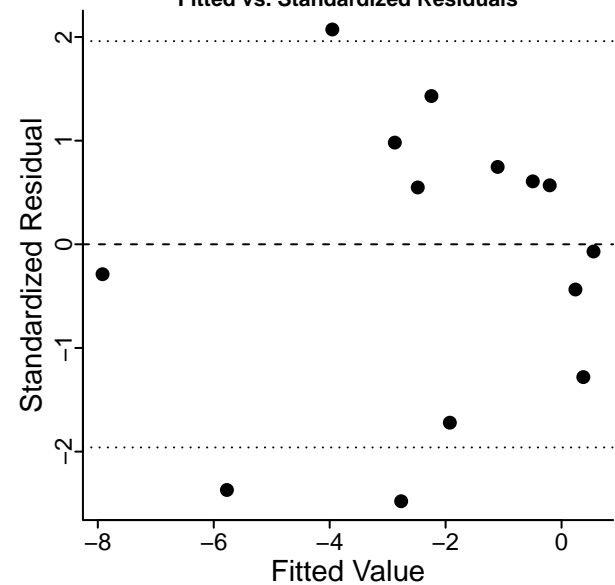

Standardized Residuals

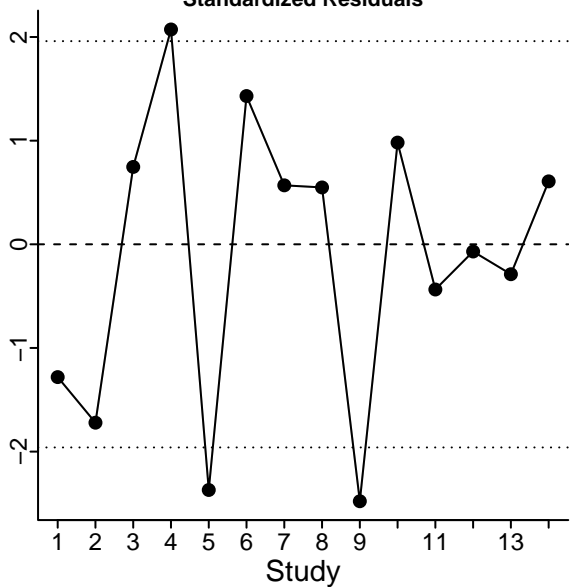

Supplement: Supplementary file 6 [file Image6.pdf]
